# Bifunctional Metal–Organic Framework-Coated Porous Hydroxyapatite Particles for Antibacterial Activity and Toluene Adsorption

**DOI:** 10.3390/ma19214690

**Published:** 2026-11-04

**Authors:** Youn-Sub Jeong, Jae Won Jeong, Jung Woo Lee, Young-Tae Kwon

**Affiliations:** 1 New-Functional Powder Materials Research Center, Korea Institute of Materials Science, Changwon 51508, Republic of Korea; aa45792@kims.re.kr (Y.-S.J.); jeongjw1204@kims.re.kr (J.W.J.); 2 Department of Materials Science and Engineering, Pusan National University, Busan 46278, Republic of Korea

**Keywords:** hydroxyapatite, metal–organic framework, antibacterial performance, toluene adsorption, ultrasonic spray pyrolysis process

## Abstract

**Highlights:**

- MOF-coated porous hydroxyapatite particles were developed for antibacterial activity and toluene adsorption.
- The composite achieved >99.9% bacterial reduction and >99% toluene removal within 30 min.
- The composite enables simultaneous control of biological and chemical air pollutants.

**Abstract:**

The simultaneous occurrence of biological and chemical pollutants in air environments presents a significant challenge for effective air purification. Herein, we developed a bifunctional composite consisting of metal–organic framework (MOF)-coated porous hydroxyapatite (Hap) particles for simultaneous antibacterial activity and toluene adsorption. Microporous Hap particles were fabricated via an atomized aerosol reaction, followed by conformal growth of a MIL-100(Fe) layer to provide adsorption sites for aromatic volatile organic compounds. A thin MIL-100(Fe) coating layer was formed via precursor adsorption during stepwise growth metal–ligand coordination layer was additionally introduced to enhance the toluene adsorption performance of the porous Hap. The resulting composite exhibited strong antibacterial activity against *Salmonella typhimurium*, *Pseudomonas aeruginosa*, and *Staphylococcus aureus*, achieving greater than 99.9% bacterial reduction. In addition, the composite showed greater than 99% toluene removal within 30 min. These results demonstrate that the porous Hap framework and MOF coating provide complementary antibacterial and VOC adsorption functions within a single material platform. The developed composite offers a promising approach for the simultaneous removal of biological and chemical pollutants and may be applicable to air purification in indoor, industrial, and medical environments.

## 1. Introduction

Rapid industrialization and urbanization have significantly deteriorated air quality by increasing the coexistence of biological and chemical pollutants in atmospheric environments. Modern air environments are commonly contaminated by microorganisms such as bacteria and viruses, as well as volatile organic compounds (VOCs) released from various chemical processes, including paints, adhesives, and petrochemical industries [1,2]. Bacteria exert both beneficial and harmful effects on the human body [3,4]. While specific bacterial species contribute to metabolic and immunological regulation, pathogenic bacteria are responsible for a broad spectrum of infectious diseases, ranging from common respiratory infections to severe systemic disorders. Although antibacterial agents such as antibiotics and antiviral vaccines have been widely used to suppress microbial infections, improper or excessive administration may induce adverse side effects or accelerate the development of antibiotic resistance. Thus, it is essential to develop material-based antibacterial strategies that can fundamentally inhibit the initial invasion, attachment, and subsequent proliferation of pathogenic microorganisms [5,6]. In addition to microbial contamination, chemical pollutants such as volatile organic compounds (VOCs) are also recognized as hazardous airborne species [7,8,9]. Among VOCs, toluene is particularly notable for its high volatility and neurotoxic properties, and chronic exposure has been linked to respiratory irritation, neurological impairment, and broader health concerns. In insufficiently ventilated indoor environments, continuous emission sources combined with limited air circulation can further elevate VOC concentrations, thereby emphasizing the need for efficient VOC removal technologies and advanced air purification materials [10]. Accordingly, the development of materials that can simultaneously mitigate both biological and chemical airborne pollutants has become an important research objective.

Metal-ion-based antibacterial materials, including Ag^+^- and Cu^2+^-containing systems, have been extensively investigated owing to their strong bactericidal activity [11,12,13]. However, limitations such as ion-related cytotoxicity, high cost, and the potential development of bacterial resistance upon repeated exposure remain significant barriers to practical implementation [14,15]. To overcome these drawbacks, alternative antibacterial materials with improved biocompatibility and chemical stability have attracted increasing interest. Hydroxyapatite (Hap), which exhibits structural and compositional similarity to the mineral phase of natural bone, has emerged as a promising candidate owing to its excellent biocompatibility, chemical stability, and low toxicity [16,17]. Unlike metal-ion-based materials, the initial antibacterial activity of Hap is triggered by rapid electrostatic entrapment and surface contact between surface-exposed Ca^2+^ ions and negatively charged bacterial membranes. Following this initial close contact, localized ion release and interfacial microenvironment changes act synergistically, leading to the disruption of ionic homeostasis, increased membrane permeability, and eventual cell death [18]. Furthermore, high-surface-area porous Hap architectures intensify bacteria–surface interactions and facilitate efficient mass transport, thereby sustaining localized antibacterial activity and enhancing overall efficacy.

Meanwhile, the removal of aromatic VOCs such as toluene from air remains a challenging task. Conventional adsorbents including activated carbon, zeolites, and silica-based materials often suffer from limited selectivity toward aromatic molecules and insufficient structural stability, thereby restricting further improvements in adsorption performance [8,19,20]. Metal–organic frameworks (MOFs) have attracted considerable attention due to their unique structural characteristics. Recent studies have further demonstrated that functional MOF-based composite systems can simultaneously address multiple air pollutants through synergistic design strategies [21,22,23]. These crystalline porous materials consist of metal ions or clusters coordinated with organic linkers and exhibit exceptionally high surface areas and tunable chemical functionalities [24,25,26]. Such features enable strong affinity toward VOC molecules through π–π interactions, coordinative bonding at exposed metal sites, and pore–molecule size matching, resulting in superior VOC selectivity compared with conventional adsorbents [27]. MIL-100 (Fe), distinguished by its mesoporous cages, exhibits pore dimensions compatible with the kinetic diameter of toluene, enabling efficient diffusion throughout the framework. Moreover, π–π stacking interactions between the aromatic BTC linkers and toluene molecules further reinforce adsorption strength and selectivity [28]. However, despite their excellent adsorption performance, bulk MOF powders often suffer from high material cost and practical difficulties associated with large-scale deployment. Therefore, reducing MOF consumption while preserving surface functionality has emerged as an important design strategy for practical air purification systems.

Herein, we develop a bifunctional Hap/MIL-100 (Fe) composite via direct in situ growth of MIL-100(Fe) on porous Hap, integrating antibacterial functionality with selective toluene adsorption. The Hap framework, produced by ultrasonic aerosol-assisted pyrolysis, features a porous architecture of high surface area that enables efficient precursor infiltration and uniform MOF nucleation. The aerosol pyrolysis process is particularly beneficial for porous material fabrication, as it allows precise control over particle morphology and pore architecture via droplet-mediated self-assembly [29]. Surface-exposed Ca^2+^ ions further coordinate with Fe^3+^ centers, facilitating the formation of a continuous MIL-100(Fe) layer. Rather than relying on bulk MOF loading, the present study introduces MIL-100(Fe) as a conformal functional coating that maximizes adsorption efficiency with minimal MOF usage. This hybrid architecture spatially separates antibacterial and adsorption functions, where porous Hap provides biocompatible antibacterial activity and mechanical stability, while the MIL-100(Fe) surface layer delivers selective aromatic VOC capture. This synergistic design overcomes the intrinsic limitations of single-component materials and establishes a versatile platform for next-generation air purification.

## 2. Materials and Methods

### 2.1. Materials

Calcium nitrate tetrahydrate (Ca(NO_3_)_2_·4H_2_O, ACS reagent, 99%) and Thioglycolic acid (C_2_H_4_O_2_S, 98%) were purchased from Sigma-Aldrich (St. Louis, MO, USA). Phosphoric acid (H_3_PO_4_, Extra Pure, 99.9%), polyethylene glycol #4000 (H(OCH_2_CH_2_)_n_OH), anhydrous ethyl alcohol (C_2_H_5_OH, 99%), and absolute ethyl alcohol (C_2_H_5_OH, 94.5%) were obtained from Samchun Chemical Co., Ltd. (Seoul, Republic of Korea). 1,3,5-Benzenetricarboxylic acid (C_9_H_6_O_6_, 98%) and iron(III) chloride hexahydrate (FeCl_3_·6H_2_O, 99+%) were purchased from Thermo Scientific (Waltham, MA, USA).

### 2.2. Manufacturing for Hap and Porous Hap

Hap particles were synthesized using an ultrasonic spray pyrolysis (USP) process. Aqueous precursor solutions were prepared by dissolving calcium nitrate tetrahydrate (Ca(NO_3_)_2_·4H_2_O) and phosphoric acid (H_3_PO_4_) in deionized water. For the synthesis of pure Hap, a stoichiometric precursor solution was prepared by mixing Ca(NO_3_)_2_·4H_2_O (1.0 M) and H_3_PO_4_ (0.6 M) to achieve a Ca/P molar ratio of 1.67. For polymer-assisted Hap synthesis, polyethylene glycol (PEG, 0.025 M) was directly added as a pore-forming and structure-directing agent to a precursor solution containing Ca(NO_3_)_2_·4H_2_O (0.5 M) and H_3_PO_4_ (0.3 M). The prepared precursor solution (100 mL) was continuously fed into the reactor tube at a controlled flow rate of 0.4 mL·min^−1^ using a syringe pump. The solution was ultrasonically atomized to generate fine aerosol droplets, which were subsequently transported into the USP reactor by an air carrier gas at a flow rate of 3 L·min^−1^. The USP reactor was equipped with two sequential heating zones to control droplet drying, decomposition, and crystallization. In the first heating zone (200 °C), solvent evaporation occurred along with gelation and precursor structure formation of Ca–P–PEG complexes. In the second heating zone (800 °C), thermal decomposition of nitrates and polymers was induced, accompanied by crystallization and structural reorganization of hydroxyapatite. During this two-step thermal treatment, gaseous species such as H_2_O and CO_2_ were released, and spherical porous Hap-based particles were formed through reconstruction of the Ca–P network. The as-formed Hap and PEG–Hap powders were collected at the reactor outlet using filter paper, and a portion of the samples was reserved for further characterization.

### 2.3. Manufacturing for Porous Hap/MOF

For MOF coating, 0.5 g of porous hydroxyapatite powder was dispersed in 99% ethanol containing thioglycolic acid (7.5 mM) and stirred at room temperature for 24 h to achieve surface modification. After completion of the reaction, the product was washed by centrifugation (7000 rpm, 5 min) five times using 94.5% ethanol to remove unreacted organic species and residual reagents. Subsequently, FeCl_3_·6H_2_O was dissolved in 99% ethanol to prepare a 0.16 M solution. The surface-modified Hap powder was added to this solution and stirred for 15 min to promote adsorption of Fe^3+^ ions. The resulting product was collected by centrifugation at 7000 rpm for 5 min and washed five times with 94.5% ethanol. For incorporation of the organic linker, 1,3,5-benzenetricarboxylic acid (BTC, 0.16 M) was dissolved in 99% ethanol and mixed with the Fe-treated Hap powder, followed by stirring for 30 min. The same centrifugation and washing procedures were then applied. The sequential Fe^3+^ adsorption and BTC coordination steps were repeated five times to ensure sufficient growth and uniform formation of the MIL-100(Fe) coating layer on the porous Hap surface.

### 2.4. Characterization

The surface and morphological features of the prepared powders were investigated using field-emission scanning electron microscopy ((FE-SEM; JSM-7001F, JEOL Ltd., Akishima, Tokyo, Japan). High-resolution transmission electron microscopy (HR-TEM; Talos F200X, FEI Company, Hillsboro, OR, USA) was employed to examine the detailed nanostructure and to verify the presence of the MIL-100(Fe) coating layer on the Hap surface. The phase composition and crystallinity were analyzed by an X-ray diffractometer (XRD; SmartLab 9 kW, Rigaku Corporation, Akishima, Tokyo, Japan). Brunauer–Emmett–Teller (BET; Micromeritics Instrument Corporation, Norcross, GA, USA) analysis was conducted to determine the specific surface area of the synthesized Hap particles. Changes in the functional groups of Hap and MOF-coated Hap particles were characterized with Fourier transform infrared spectroscopy (FT-IR; Nicolet iS10, Thermo Fisher Scientific, Madison, WI, USA). High-performance X-ray Photoelectron Spectrometer (XPS; ESCALAB 250, Thermo VG Scientific, East Grinstead, UK) was utilized to identify the formation of MIL-100(Fe) on the surface of Hap particles. The antibacterial activity of the synthesized powders was quantitatively evaluated according to the ASTM E2149-20 standard [30] method using dynamic contact conditions. The antibacterial activity was evaluated by FITI Testing & Research Institute (Seoul, Republic of Korea) using five reference bacterial strains: *Salmonella typhimurium* (KTTC 1925; Initial concentration: 1.1 × 10^5^ CFU/mL), *Pseudomonas aeruginosa* (ATCC 27853; Initial concentration: 1.0 x 10^5^ CFU/mL), *Staphylococcus aureus* (ATCC 6538; Initial concentration: 1.2 × 10^5^ CFU/mL), *Klebsiella pneumoniae* (ATCC 4352; Initial concentration: 1.1 × 10^5^ CFU/mL), and *Escherichia coli* (ATCC 8739; In-itial concentration: 1.3 × 10^5^ CFU/mL). The ATCC and KCTC designations correspond to the American Type Culture Collection (Manassas, VA, USA) and the Korean Collection for Type Cultures (Jeongeup, Republic of Korea), respectively. Prior to testing, all powder samples (1.0 g) were sterilized by autoclaving at 121+/−2 °C for 15 min. Each sterilized sample (1.0 g) was immersed in 50 mL of phosphate-buffered solution (pH 7.2). The mixture was continuously shaken and incubated at 37 °C for 24 h to ensure uniform dynamic contact between the particles and bacterial cells. After incubation, serial dilutions were performed, and viable bacteria were enumerated using the standard agar plate colony counting method. A control group without powder treatment was tested under identical conditions. All measurements were conducted in triplicate (*n* = 3), and the bacterial reduction rate (R, %) was calculated using the equation: R (%) = [(A − B)/A] × 100, where A and B represent the viable bacterial counts (CFU/mL) of the control and the treated sample after 24 h of incubation, respectively. For gas adsorption experiments, 0.5 g of the powder was placed in a 0.5 L Tedlar bag containing 50 ppm toluene (C7H8) in air. The concentration of toluene in the bag was monitored to evaluate the adsorption performance of the porous Hap/MOF composite. The adsorption performance was further evaluated using a calibrated toluene gas detector tube (GV-100S, GASTEC Co., Ltd., Ayase, Japan).

## 3. Results

### 3.1. Fabrication of Porous Hap/MIL-100(Fe) Composites

**Figure 1:**
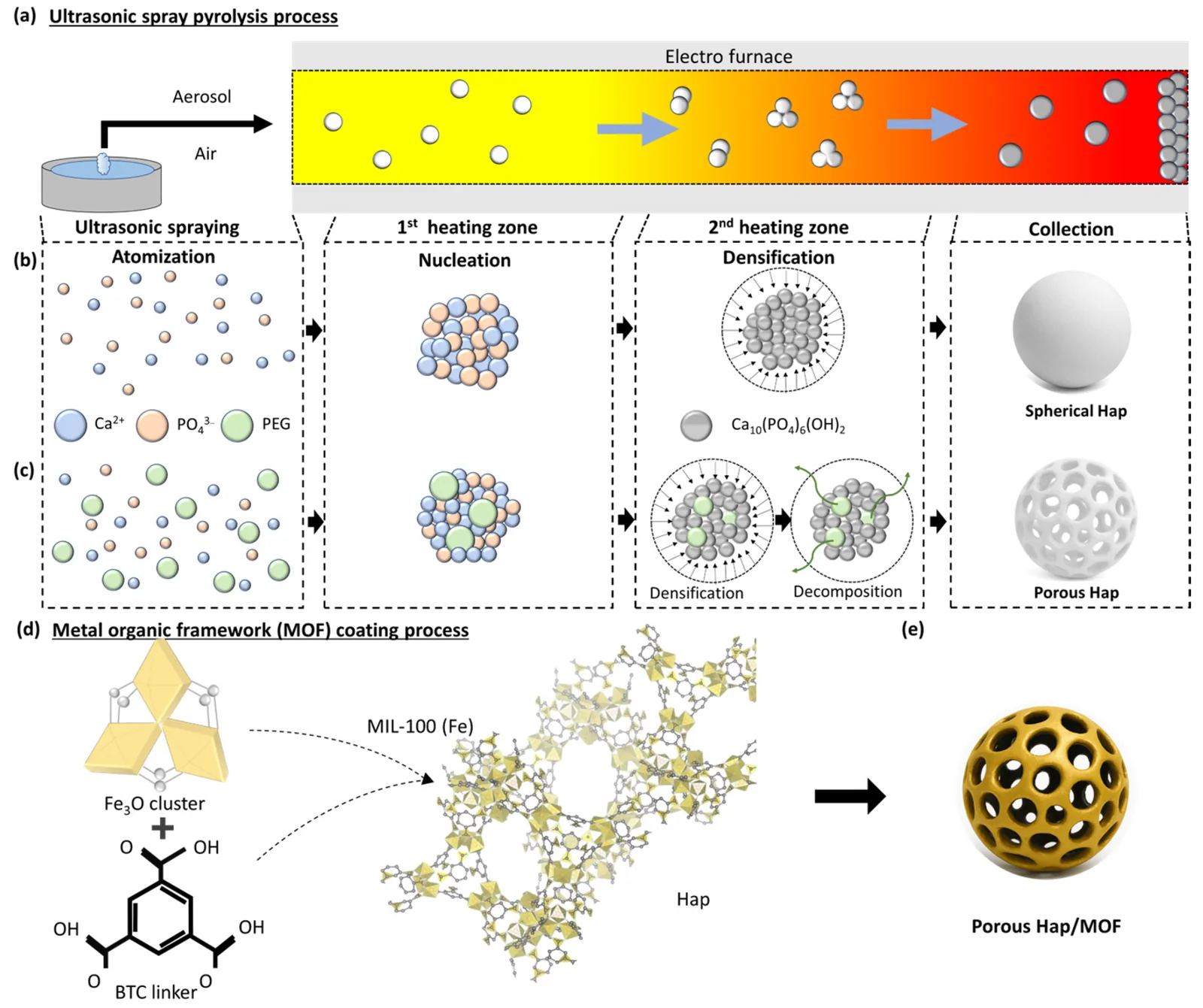
(**a**) Schematic illustration of the ultrasonic-assisted spray pyrolysis (USP) process for fabricating spherical and porous Hap particles, prepared with and without PEG. The USP system consists of four sequential zones: ultrasonic atomization, primary heating, secondary heating, and particle collection. (**b**) Formation of dense spherical Hap particles via aerosol-based reaction of calcium and phosphate precursors. (**c**) Formation of porous spherical Hap particles synthesized in the presence of PEG as a pore-forming additive. (**d**) Illustration showing the MIL-100(Fe) coating formed on the surface of a porous Hap particle. Surface-anchored Fe^3+^ ions coordinate with BTC to form Fe_3_O clusters, driving MIL-100(Fe) growth on Hap. (**e**) Schematic of the final porous Hap/MOF morphology.

Figure 1a illustrates the USP process employed for the synthesis of spherical and porous Hap powders under controlled precursor conditions. The aqueous precursor solution is converted into fine aerosol droplets through ultrasonic atomization and subsequently transported into a tubular electric furnace consisting of sequential heating zones. When the aqueous aerosol droplets enter the first heating zone at 200 °C, rapid solvent evaporation induces the nucleation of Hap precursors. The precursors subsequently undergo thermal decomposition and crystallization in the high-temperature zone at 800 °C to form crystalline Hap particles. During this process, the reaction pathway remains confined within individual droplets, promoting micro-scale compositional uniformity while minimizing concentration gradients between droplets. Each droplet acts as an independent microreactor, providing a homogeneous thermal and chemical environment and enabling the continuous production of spherical particles with high reproducibility. Such intrinsic characteristics of the USP process are advantageous for large-scale production and industrial scalability.

Figure 1b schematically illustrates the particle formation mechanism of Hap powders prepared from a precursor solution containing calcium nitrate and phosphoric acid. Each droplet corresponds to a single final particle, and the droplet size therefore determines the resulting particle size. Rapid solvent evaporation increases the concentrations of Ca^2+^ and PO_4_^3−^ ions within the droplet. Once supersaturation is reached, homogeneous nucleation of amorphous calcium phosphate occurs throughout the droplet. The particles subsequently pass through the second heating zone, where densification proceeds and relatively smooth and dense spherical Hap particles are produced. The overall reaction can be expressed as follows:

Ca(NO_3_)_2_·4H_2_O → Ca(OH_2_)_6_^2+^ (aq) + 2NO_3_^−^ (aq)

H_3_PO_4_ → 3H^+^ (aq) + PO_4_^3−^ (aq)

10Ca^2+^ (aq) + 6PO_4_^3−^ (aq) + 2OH^−^ (aq) → Ca_10_(PO_4_)_6_(OH)_2_ (s).

When PEG is incorporated into the precursor solution (Figure 1c), thermal decomposition of PEG occurs in the second heating zone, generating gaseous byproducts. The evolution of these gases within the partially solidified inorganic matrix during particle densification induces the formation of internal pores. Although the overall spherical morphology is preserved, porous Hap particles with well-developed internal voids are formed. In this case, densification and polymer decomposition occur simultaneously, resulting in a structure distinct from that of dense Hap particles. Both dense and porous Hap particles are ultimately collected as spherical particles at the final stage of the process. Figure 1d depicts the formation process of the MIL-100(Fe) coating on the surface of porous Hap. Fe_3_O clusters and BTC linkers coordinate to form the MIL-100(Fe) framework, which grows on the surface of porous Hap. The Hap surface provides active sites for Fe species adsorption and heterogeneous nucleation, promoting conformal MOF growth along the particle surface. Unlike bulk crystallization, which produces isolated MOF crystals, this approach leads to the formation of a continuous MOF coating layer intimately integrated with the Hap ceramic core. Rather than maximizing MOF loading, the present design intentionally employs an ultrathin conformal MIL-100(Fe) layer to introduce selective adsorption functionality while preserving the intrinsic antibacterial activity and structural robustness of the Hap core. Figure 1e shows the final porous Hap/MOF composite structure, consisting of a porous Hap core coated with an outer MIL-100(Fe) layer. This hierarchical architecture simultaneously ensures the mechanical stability of the porous ceramic framework and introduces additional surface functionality through the MOF layer. Such an integrated structure is expected to provide synergistic advantages in antibacterial activity and VOC adsorption, as systematically evaluated in the subsequent sections.

### 3.2. Structural Characterization of Hap/MIL-100(Fe) Composites

**Figure 2:**
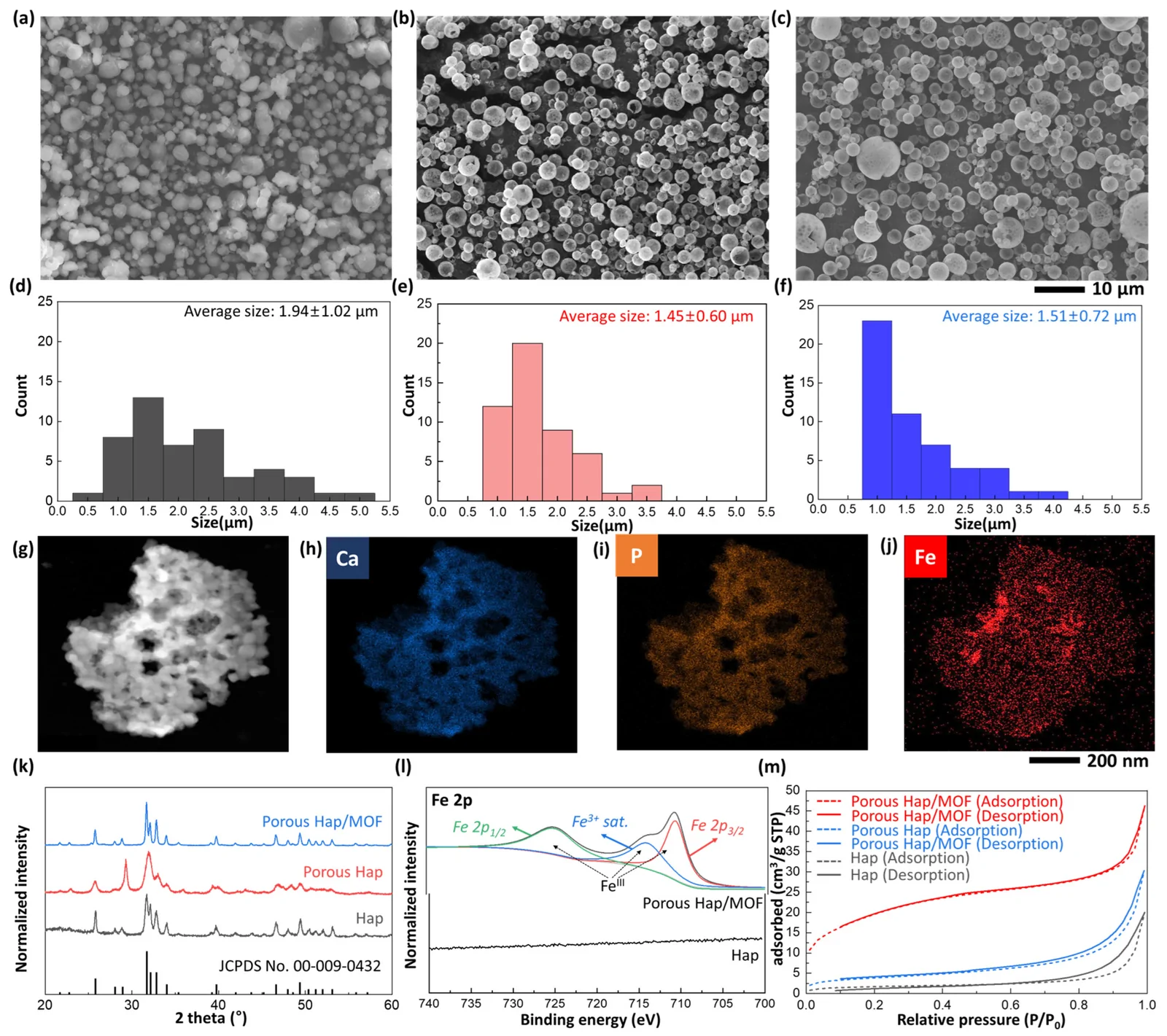
(**a**–**c**) SEM images of (**a**) spherical Hap, (**b**) porous Hap, and (**c**) porous Hap/MOF particles. (**d**–**f**) Particle size distributions corresponding to (**d**) spherical Hap, (**e**) porous Hap, and (**f**) porous Hap/MOF particles. (**g**) TEM images and the corresponding elemental mapping of (**h**) calcium, (**i**) phosphorus, (**j**) iron in porous Hap/MOF particle. Characterization results of porous Hap/MOF particles obtained using (**k**) XRD analysis and (**l**) Fe 2p XPS spectra. (**m**) Nitrogen adsorption–desorption isotherms for BET surface area analysis.

**Figure 3:**
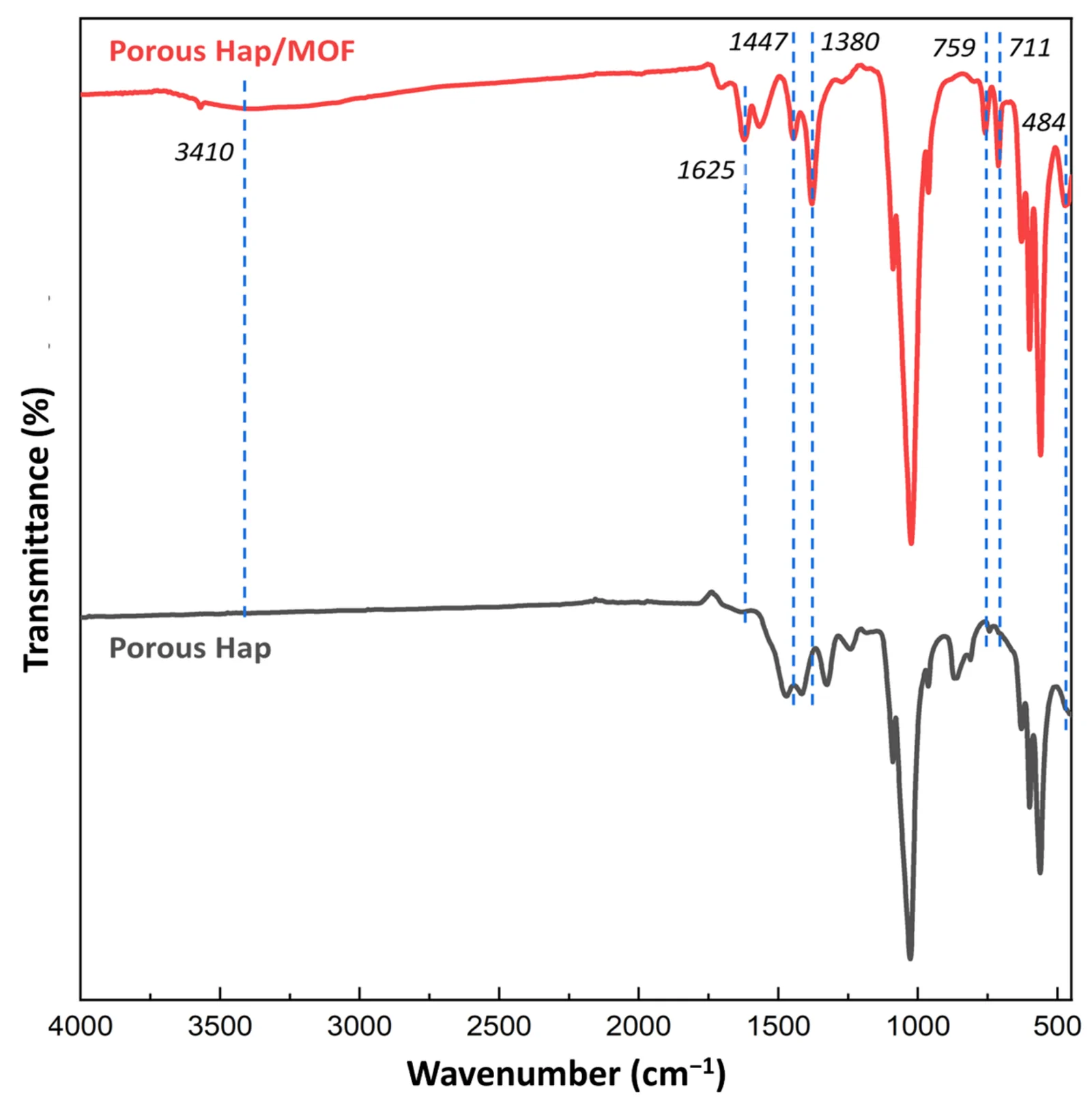
FT-IR spectra of porous Hap (black line) and porous Hap/MOF (red line) particles.

Figure 2 presents the morphology and structural characteristics of dense Hap, porous Hap, and porous Hap/MOF particles. Dense Hap exhibits relatively smooth surfaces with limited porosity (Figure 2a). In contrast, porous Hap shows increased surface roughness and clearly visible pores (Figure 2b). These morphological features confirm that gas-templated pore formation occurred during the USP process. The MOF-coated sample retains a spherical morphology with slightly textured surfaces, as shown in Figure 2c. No distinguishable MOF crystals are observed in the SEM images, indicating that the MOF layer is extremely thin and uniformly covers the particle surface. The absence of discrete MOF crystals suggests surface-confined growth rather than bulk MOF formation, supporting the intended low-loading coating strategy. Figure 2d–f show the particle size distributions determined by counting 50 particles for each sample. Particle size distributions remain narrow across all samples, which indicates uniform droplet formation during the USP process. In Figure 2d,e, the slight reduction in mean particle size for porous samples is attributed to the decrease in effective density caused by internal void formation. Such monodisperse particle characteristics improve the reproducibility of adsorption kinetics by minimizing diffusion variability associated with particle size differences. The particle size distribution remains nearly unchanged after MOF coating, indicating that the coating layer is sufficiently thin and does not significantly affect the overall particle size (Figure 2f). TEM and EDS analysis (Figure 2g–j and Supplementary Figure S1) demonstrate a conformal MOF coating on the porous Hap particles. The elemental mapping of calcium (Figure 2h) and phosphorus (Figure 2i) confirms the presence of the porous Hap core, whereas the homogeneous distribution of iron (Figure 2j) reveals that the MOF layer covers the particle surface continuously with a thickness of approximately 10–15 nm. Such a homogeneous Fe distribution indicates that adsorption sites are well-dispersed across the particles 26.8°, 32.5°, 33.1°, and 34.6°, which are consistent with the standard hydroxyapatite phase (JCPDS No. 00-009-0432). No additional diffraction peaks are observed after the formation of porous structures or MOF coating. This result indicates that neither the introduction of porosity nor the ultrathin MOF layer significantly alters the crystalline structure of Hap, which can be attributed to the extremely thin MOF coating layer. XPS was used to analyze the surface composition and chemical states of the prepared porous Hap/MOF particles (Figure 2l). The uncoated porous Hap sample shows no Fe 2p signal, whereas the MOF-coated sample exhibits clear Fe 2p peaks. The high-resolution Fe 2p spectrum can be deconvoluted into Fe 2p3/2 and Fe 2p1/2 peaks at 710.9 and 725.4 eV corresponding to Fe(III), along with a satellite peak at 714.4 eV associated with Fe(III) [31,32]. These results confirm the successful formation of the MOF layer on the Hap particles. In Figure 2m, nitrogen adsorption–desorption isotherms reveal a significant difference in surface area among dense Hap, porous Hap, and porous Hap/MOF samples. FT-IR spectroscopy was employed to identify the functional groups of MIL-100(Fe) coated on Hap particles in the range of 4000–450 cm^−1^ (Figure 3). The FT-IR spectrum of the MIL-100(Fe) coating layer exhibited characteristic absorption bands assigned to -OH stretching (3410 cm^−1^), C=O stretching (1625 cm^−1^), -OH vibration (1447 cm^−1^), C-O stretching (1380 cm^−1^), and aromatic C=C stretching (759 and 711 cm^−1^), confirming the presence of the H_3_BTC ligand. In addition, the absorption band at 484 cm^−1^ is attributed to the Fe-O stretching vibration, indicating coordination between oxygen atoms and Fe ions [31,32,33]. The incorporation of PEG in the USP process increases the BET surface area from 5.82 m^2^/g for dense Hap to 13.49 m^2^/g for porous Hap. Upon subsequent MOF coating, the BET surface area further increases to 69.52 m^2^/g for the porous Hap/MOF sample, indicating an additional contribution of the MOF layer to the overall surface area. The BET surface area and pore volume is summarized in Table 1. The corresponding pore volumes are 0.03423 cm^3^/g for dense Hap, 0.05159 cm^3^/g for porous Hap, and 0.70438 cm^3^/g for porous Hap/MOF, showing a continuous increase in pore volume with both porosity development and MOF coating. In addition, the increase in adsorption at high relative pressures within the hysteresis loop indicates the contribution of macropores, which is consistent with the presence of macro-porous structures observed in the SEM images of the Hap/MOF sample. However, it should be noted that the overall BET surface area of the porous Hap/MOF composite remains relatively low compared to that of bulk MIL-100(Fe), which typically exceeds 1000 m^2^/g. This can be attributed to the thin MOF coating layer formed on the Hap surface, resulting in limited MOF loading, as well as possible surface coverage and partial pore blocking during MOF growth. Despite the relatively low surface area, the presence of accessible active sites from MIL-100(Fe), particularly on the external surface and at the interface, can effectively contribute to toluene adsorption. This intentionally limited surface area reflects the design strategy of incorporating MIL-100(Fe) as a functional interfacial layer rather than as a high-capacity standalone adsorbent. Such a configuration reduces material consumption while still providing sufficient active sites for VOC adsorption.

### 3.3. Antibacterial Performance of Hap/MIL-100(Fe) Composites

**Figure 4:**
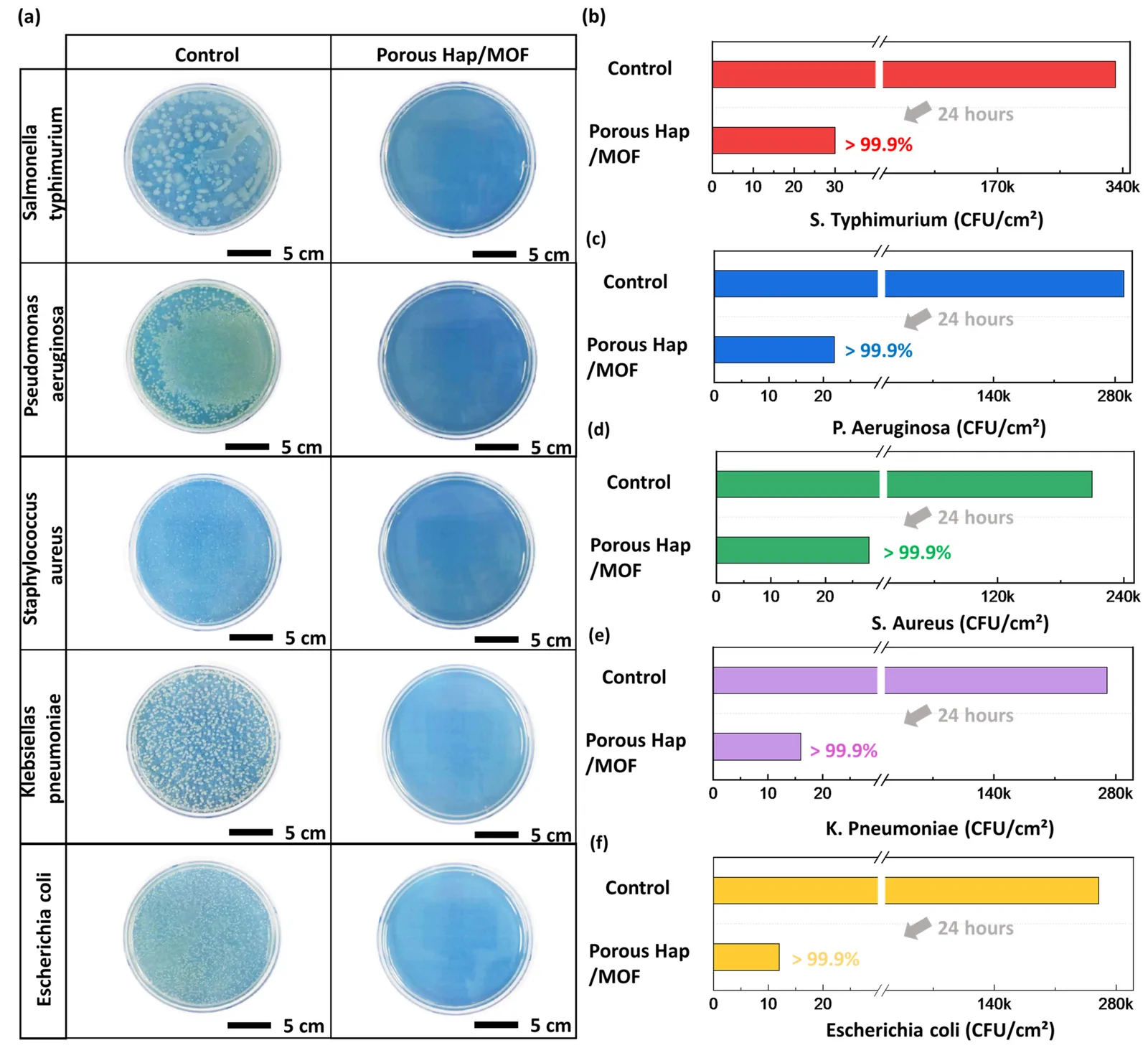
(**a**) Photographs of colony-forming unit assays against *Salmonella typhimurium*, *Pseudomonas aeruginosa*, *Staphylococcus aureus*, *Klebsiella pneumoniae*, and *Escherichia coli*. Left images present the original bacterial colonies (control sample), whereas right photos show the cell array exposed to Porous Hap/MOF. (**b**–**f**) Quantitative analysis of viable bacterial counts for each (**b**) *Salmonella typhimurium*, (**c**) *Pseudomonas aeruginosa*, (**d**) *Staphylococcus aureus*, (**e**) *Klebsiella pneumoniae*, and (**f**) *Escherichia coli* after 24 h of incubation.

Figure 4 illustrates the antibacterial activities of Hap particles against five representative bacterial strains (*S. typhimurium*, *P. aeruginosa*, *S. aureus*, *K. pneumoniae*, and *E. coli*), which are widely used as model strains due to their clinical relevance and distinct cell wall structures. Testing against these species enables evaluation of antibacterial performance against both Gram-positive (*S. aureus*) and Gram-negative bacteria (*S. typhimurium*, *P. aeruginosa*, *K. pneumoniae*, and *E. coli*). The antibacterial performance of the manufactured porous Hap/MOF particles was first assessed qualitatively. Figure 4a demonstrates the complete elimination of visible bacterial colonies compared with the control group. The culture medium treated with porous Hap/MOF remains transparent, indicating effective inhibition of bacterial growth. The quantitative analysis of viable bacterial counts (Figure 4b–f) confirms the high antibacterial activity of porous Hap/MOF particles. After 24 h of incubation, more than 99.9% of bacterial cells, including *S. typhimurium*, *P. aeruginosa*, *S. aureus*, *K. pneumoniae*, and *E. coli*, were eliminated. Despite the thin MOF coating, Hap particles exhibit intrinsic antibacterial properties governed by a cooperative multi-step process. Initially, electrostatic attraction ensures close contact between bacteria and the porous Hap surface; subsequently, the localized release of Ca^2+^, PO_4_^3−^, and OH^−^ ions at this intimate interface effectively disrupt bacterial membrane integrity and increases membrane permeability, ultimately leading to cell death without requiring high bulk leaching. The preservation of antibacterial activity after MOF coating confirms that the ultrathin MIL-100(Fe) layer does not block ion release from Hap, demonstrating functional compatibility between the ceramic core and MOF shell. In our previous study, dense Hap exhibited approximately 97% antibacterial activity against *S. aureus*, *K. pneumoniae*, and *E. coli* [34]. The enhanced antibacterial performance observed for porous Hap in the present study suggests that porosity affects antibacterial activity not only through surface chemical composition but also through structural factors. Specifically, compared with dense Hap, porous Hap exhibits increased surface roughness and well-developed pore structures, which increase the probability of contact between bacterial cells and particle surfaces. This porous architecture effectively enlarges the interfacial contact area between particles and bacteria, thereby facilitating closer interactions at the particle–cell interface.

In addition to antibacterial performance, the biocompatibility of the constituent materials is an important consideration for their potential biological applications. El-Bassyouni et al. [35] investigated the in vitro cytotoxicity of hydroxyapatite (Hap) nanoparticles and reported favorable cell viability under the tested conditions, demonstrating their potential cytocompatibility. Similarly, Chen et al. [36] systematically evaluated the cytotoxicity of MIL-100(Fe) using normal human liver cells (HL-7702) and hepatocellular carcinoma cells (HepG2). At a concentration of 80 μg/mL, cell viability exceeded 85.97% and 91.20%, respectively, indicating low cytotoxicity under the investigated conditions. These findings demonstrate that both Hap and MIL-100(Fe) exhibit favorable cytocompatibility within specific experimental conditions. Together with the antibacterial performance demonstrated in the present study, these previously reported findings provide valuable insights into the biological properties of the constituent materials and highlight the potential of Hap/MIL-100(Fe) composites as multifunctional hybrid materials.

### 3.4. Toluene Adsorption Performance of Hap/MIL-100(Fe) Composites

**Figure 5:**
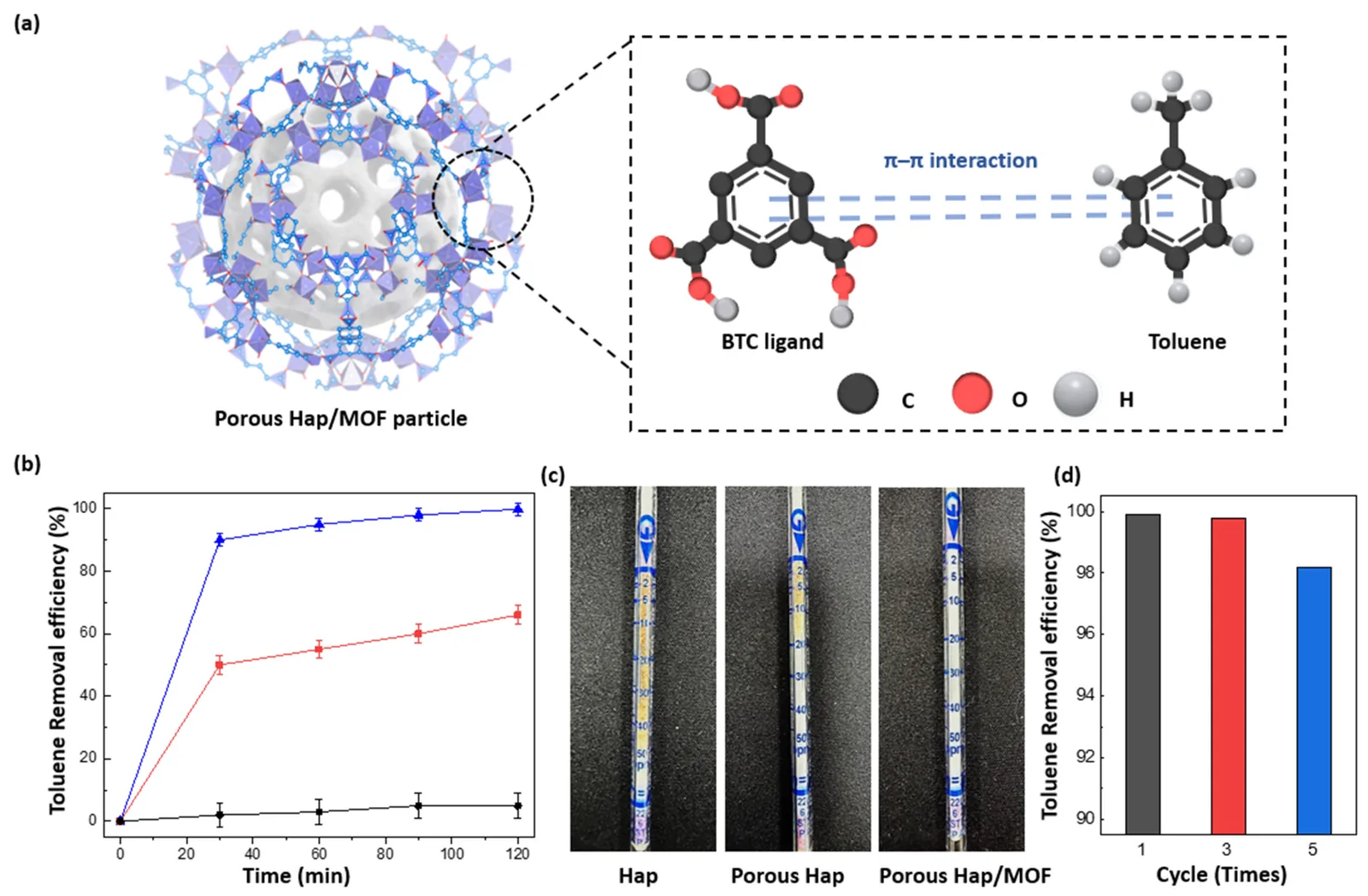
(**a**) Schematic illustration depicting the adsorption mechanism of toluene through π–π interactions between the aromatic BTC ligands in the MIL-100(Fe) layer and toluene molecules. (**b**) Toluene removal efficiency of Hap, porous Hap, and porous Hap/MOF samples as a function of exposure time, as determined by gas detector tubes. (**c**) Photographs of toluene gas detector tube results after exposure to gas bags containing Hap, porous Hap, and porous Hap/MOF particles following toluene adsorption. (**d**) Reproducibility test of toluene removal efficiency measured after the 1st, 3rd, and 5th adsorption cycles.

Figure 5a illustrates the toluene adsorption mechanisms for the porous Hap/MOF particle. Among various MOF candidates, MIL-100(Fe), composed of Fe_3_O clusters and BTC ligands, was employed for toluene adsorption. The adsorption of toluene on MIL-100(Fe) is primarily governed by π–π interactions between the aromatic rings of toluene molecules and the benzene rings of the BTC ligand in the framework. These interactions provide strong affinity and selectivity toward aromatic VOCs. In addition, the Fe_3_O clusters in MIL-100(Fe) contain coordinatively unsaturated Fe sites that can interact with the π-electron cloud of toluene. Although Fe- π interaction is weaker than π–π interactions, it contributes to the overall adsorption capacity and stability of the adsorbed molecules. The toluene removal performance was evaluated by placing dense Hap, porous Hap, and porous Hap/MIL-100(Fe) particles in a 0.5 L Tedlar bag containing 50 ppm toluene at 25 °C and 40% relative humidity (RH), followed by measuring the residual toluene concentration after 30, 60, 90, and 120 min. In Figure 5b, the bag containing dense Hap particles exhibited a low toluene removal efficiency of less than 10%, whereas porous Hap achieved approximately 60% removal due to its increased specific surface area. Porous Hap/MOF achieved over 99% toluene removal, reflecting the enhanced chemical affinity provided by the MOF coating. The data shown in Figure 5b represent the average values obtained from three independent measurements, and error bars corresponding to the standard deviation have been included to demonstrate reproducibility. In addition, the adsorption capacity was estimated based on the decrease in toluene concentration measured using a detector tube in a 0.5 L Tedlar bag. The initial toluene concentration was 50 ppm, and 0.5 g of adsorbent was used, resulting in a maximum adsorption capacity of approximately 0.19 mg g^−1^ based on ppm-to-mass conversion. The time-dependent removal profile shows rapid adsorption kinetics, reaching approximately 90% removal within 30 min. The high initial removal efficiency suggests that adsorption on the external surface dominates during the early stage, whereas further uptake occurs through diffusion into the internal pore structure. Compared with recent MOF-based composite systems reported in the literature, the present porous Hap/MOF composite is distinguished by its bifunctional capability, enabling the simultaneous mitigation of both biological and chemical airborne pollutants within a single material platform. While many previous studies have primarily focused on VOC adsorption performance, the current system integrates effective toluene removal with inherent antibacterial functionality, highlighting its potential as a versatile air purification material under practical conditions. Figure 5c presents photographic images of gas detector tubes used to indicate the residual toluene concentration. The yellow discoloration of the indicator layer corresponds to the reaction with toluene, and the length of the colored stain reflects the remaining toluene level in the system. Reusability testing shown in Figure 5d demonstrates minimal performance degradation after five cycles. After each adsorption–desorption cycle, the sample was regenerated by heating at 100 °C for 2 h under vacuum to remove the adsorbed toluene molecules, followed by cooling to room temperature before the next cycle. The slight decrease is attributed to partial pore blockage or minor framework restructuring. However, the stability confirms that the adsorption process remains largely reversible and is governed by non-destructive interactions. The primary advantages of the antibacterial and VOC adsorption capabilities investigated in this study are summarized in Table 2, which provides a comparative analysis with recently developed materials.

## 4. Conclusions

This paper reports materials design and fabrication processes to realize bifunctions, enabling the removal of both pathogenic bacteria and VOCs such as toluene. The ultrasonic spray pyrolysis approach allows facile control of the porous structure of Hap ceramics, which enhances the reactive surface area. The porous architecture, which enhances the interfacial contact area between Hap particles and bacteria, effectively eliminated over 99.9% of five representative bacterial strains, including *S. typhimurium*, *P. aeruginosa*, *S. aureus*, *K. pneumoniae*, and *E. coli*. In this hybrid system, porous Hap primarily functions as a robust antibacterial platform, while the conformally grown MIL-100(Fe) layer provides a chemically selective adsorption domain for VOC molecules. The conformal MIL-100(Fe) layer coated on porous Hap particles exhibited high adsorption affinity for toluene, achieving >99% removal within 30 min, due to favorable π–π interactions with the aromatic rings of the organic ligands. Importantly, the integration strategy enables complementary functionality rather than simple material combination, where the porous Hap framework ensures structural robustness and antibacterial activity, and MIL-100(Fe) contributes high surface area and adsorption selectivity toward aromatic VOCs. Notably, the integration of porous Hap with MIL-100(Fe) preserved the structural stability and functional performance of both components. From a practical perspective, the aerosol-based synthesis and conformal coating approach provide a scalable and potentially cost-effective route for producing multifunctional air purification materials. Overall, the Hap/MIL-100(Fe) composite system proposed in this work represents a promising material strategy for next-generation air purification and environmental pollution control. The design concept presented herein can be extended to a wide range of MOF–ceramic hybrid systems, providing a versatile platform for the development of multifunctional materials capable of simultaneously addressing diverse airborne contaminants.

## Figures and Tables

**Table 1:** BET surface area and pore volume of synthesized materials, including Hap, porous Hap, and Porous Hap/MOF.

Materials	BET Surface Area (m^2^/g)	Pore Volume (cm^3^/g)
Hap	5.82	0.0342
Porous Hap	13.49	0.0516
Porous Hap/MOF	69.52	0.7044

**Table 2:** Comparison of antibacterial and VOC absorption properties of various composite materials.

References	Materials	Target Pollutant	Functionality	Antibacterial Property (%)	VOC (Toluene) Removal Efficiency (%)
This work	Hap-MIL-100(Fe) core–shell structure	*S. typhimurium*, *P. aeruginosa*, *S. aureus*, *K. pneumoniae*, *E. coli*, Toluene	Bifunctional	>99.9	>99
[22]	Zr-based MOFs (UiO-66, NU-1000)	*E. coli*, *S. aureus*	Monofunctional	>99.9	-
[23]	Zr-based MOFs (Zr-TSS-1)	*E. coli*, *S. aureus*	Monofunctional	>99.9	-
[37]	Cu-MOF	*E. coli*	Monofunctional	97.7	-
[38]	Fe_3_O_4_-Cu-MOF	*E. coli*, *S. aureus*	Monofunctional	>90	-
[39]	MOF (UN-66@PSB)	Toluene	Monofunctional	-	95.73
[40]	Electret filter media coated with MOF (MIL-125-NH_2_)	Toluene	Monofunctional	-	>80
[41]	Fe-MOF (MIL-88)	Toluene	Monofunctional	-	>99.9

## Data Availability

The original contributions presented in this study are included in the article/Supplementary Materials. Further inquiries can be directed to the corresponding authors.

## Supplementary Materials

The following supporting information can be downloaded at https://www.mdpi.com/article/10.3390/ma19214690/s1. Figure S1: TEM images and the corresponding elemental mapping of calcium, phosphorus, and iron in dense Hap/MOF particle.
