# Supplementary material for: Bifunctional Metal–Organic Framework-Coated Porous Hydroxyapatite Particles for Antibacterial Activity and Toluene Adsorption
Source: Materials (Basel). 2026 Nov 4;19(21):4690. doi: 10.3390/ma19214690 (PMC13765219; doi:10.3390/ma19214690)
Supplement: Supplementary file 1 [file materials-19-04690-s001.zip › materials-4644961-supplementary.pdf]

Supplementary Material

Bifunctional Metal–Organic Framework–Coated Porous Hydroxyapatite Particles for Antibacterial Activity and Toluene Adsorption

Youn-Sub Jeong[a,b], Jae-Won Jeong[a], Jung Woo Lee[b,*], and Young-Tae Kwon[a,*]

[a] New-Functional Powder Materials Research Center, Korea Institute of Materials Science, Changwon, South Korea
[b] Department of Materials Science and Engineering, Pusan National University, Busan, South Korea

* Corresponding author. New-Functional Powder Materials Research Center, Korea Institute of Materials Science, Changwon, South Korea, Department of Materials Science and Engineering, Pusan National University, Busan, South Korea.
E-mail address: ykwon87@kims.re.kr (Y.-T. Kwon), jungwoolee@pusan.ac.kr (J. W. Lee).

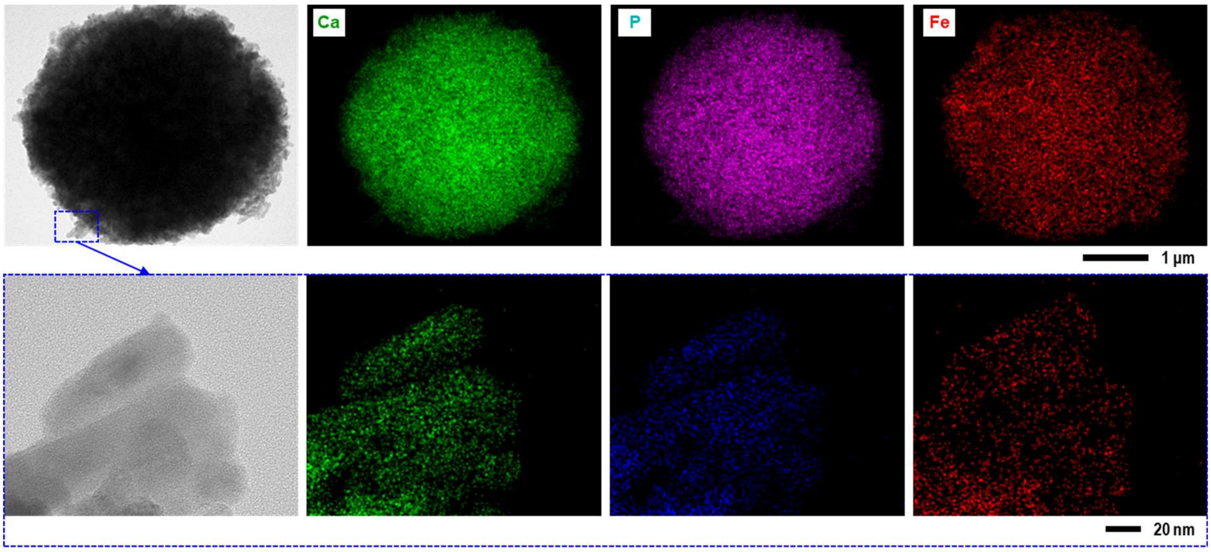

Figure S1. TEM images and the corresponding elemental mapping of calcium, phosphorus, and iron in dense Hap/MOF particle.
